# Burnout Syndrome in Brazilian Medical Doctors: A Cross-Sectional Examination of Risk and Protective Factors

**DOI:** 10.3389/frhs.2021.760034

**Published:** 2021-11-16

**Authors:** Natalia Dalla Costa Becker, Adilson Carlos da Rocha, Franciele Aní Caovilla Follador, Guilherme Welter Wendt, Lirane Elize Defante Ferreto, Paulo Nunes Fortes, João Paulo Arruda Amorim

**Affiliations:** ^1^Department of Health Sciences, Postgraduate Program in Applied Health Sciences, Francisco Beltrão, Western Paraná State University, Paraná, Brazil; ^2^Department of Social Sciences, Francisco Beltrão, Western Paraná State University, Paraná, Brazil; ^3^Department of Health Sciences, Faculty of Medicine Francisco Beltrão, Western Paraná State University, Paraná, Brazil

**Keywords:** burnout, physicians, risk factors, well-being, hospitals

## Abstract

**Objectives:** This investigation sought to identify the prevalence of Burnout Syndrome (BS) among Brazilian medical doctors (BS) and the associations with risk factors and protective factors.

**Methods:** Out of 206 registered MD from a medium-sized municipality, 121 were enrolled in this cross-sectional study. Convenience sampling was used. Based on Cohen's *f* ^2^, a power of 98% and a 0.05 alpha was achieved. MD responded to sociodemographic questions and to the Portuguese-version of the Maslach Burnout Inventory–Human Services Survey (MBI). Risk and protective factors linked with BS were examined with regression analyses.

**Results:** The age of the participants ranged from 25 to 69 years (M = 40.89; SD = 10.13) and 73.6% were male. The prevalence of BS was 7.5%. Differential aspects were related to BS. For instance, while not reporting satisfaction with the institution (β = 16.16, *p* < 0.001) and not practicing physical exercise (β = 7.39, *p* = 0.014) were associated with higher scores in the BS composite score, those who did not intend to change their careers (β = −17.15, *p* < 0.001) and participants who saw mental health specialists (β = −8.99, *p* = 0.007) scored lower, accounting for nearly a half of the BS composite score (*R*^2^ = 46%).

**Conclusion:** The prevalence of BS in this study falls within the range previously reported among healthcare professionals (i.e., 2.6–11.8%). Moreover, data suggested that commitment with the occupation and with the participant's own mental health could boost reactions against the deleterious effects of the BS. In this sense, organizations can develop strategies for preventing BS, a process that is known to be chronic and, to some extent, preventable.

## Introduction

The current configuration of work has been linked to new profiles of workers' morbidity and mortality, with intense stress and burnout syndrome (BS) as emerging examples (1). BS derives from a professional context filled with emotional stressors and a complex set of social relations which result from the professional's relation with his or her work environment (2). Indeed, BS is understood as one of the results of an imbalance in the connection between the individual and his or her work or organization, a processual condition that is hard to detect in its initial stages (3). It usually includes low personal accomplishment (PA), elevated emotional exhaustion (EE), as well as depersonalization (DE) (4).

Doctors are among the healthcare professionals with high incidence of BS. Personality traits common among them, such as perfectionism, might very frequently result in relentless, compulsive, and skeptical and cynical actions (3, 5). Moreover, social pressure on these professionals, from whom flawless and unsustainable conducts are expected, must also be considered. As a result, doctors experience weary work routines characterized by reduced spare time, scarce control over their work environments, lack of qualification to deal with patients' emotional demands, and a low degree of autonomy (3, 6, 7). Those are important risk factors associating the profession to BS (6, 8).

BS might result in absenteeism, increased costs for the employer organization, and therefore it does not only hinder the quality of the service provided, but also influences on the general productivity and profit of the institution (9). Besides, BS directly affects patients, colleagues, as well as the doctor's own work, highlighting the need of identifying not only its prevalence, but also the risk factors underpinning it. Notably, a better comprehension of BS might contribute to the planning and execution of workers' health-oriented actions.

Research on the BS in the Brazilian context has flourished over the last years, although data regarding MD are scarce and still underrepresented in studies with healthcare workers (10). Most of the past research conducted in the country have been carried out either in major cities [i.e., (11)] or focused mostly on the prevalence of the syndrome [i.e., (12)]. Equally important, studies examining the multivariate correlates of the syndrome and its subdomains among MD were not located (10–12). Consequently, the present study aimed to estimate the prevalence of BS and its associated risk and protective factors in Brazilian MD from hospitals of a specific municipality in the state of Paraná, Brazil.

A few working hypotheses were established based on the literature. First, that a larger level of EE would correspond to an increased workload, and a lower PA would be connected to a desire to change profession (11, 13). This study predicted a higher association between DE and factors of discontentment with the work environment, as well as with overload, such as the number of patients seen each day (14, 15). It has been also assumed that factors like seeing mental health professionals, a reduced number of night shifts, and lessened reports of work overload would exert a protective role toward BS (14, 16).

## Materials and Methods

### Participants and Design

This is a cross-sectional study. Its population is composed by all the doctors in Francisco Beltrão, Paraná, which amount to 206 subjects, according to data of the Regional Medical Council, subsection Francisco Beltrão, for 2018. The convenience sample included 121 respondents, 58.7% of the doctors working in hospitals in that municipality (See the inclusion criteria in the section “Procedures”). Sample size calculations were not performed *a priori* since all eligible participants were invited to take part in the study.

### Measures

The data collection was performed with the aid of a sociodemographic and occupational questionnaire accompanied by the Maslach Burnout Inventory—Human Services Survey (MBI-HSS; α = 0.75). The MBI-HSS is a 22-item inventory that evaluates the syndrome's three dimensions: EE (nine items), DE (five items), and PA (eight items) (4, 17–19). The EE dimensions measures feelings of being emotionally overwhelmed and exhaustion; DE refers to impersonal attitudes with others while at work. Finally, the PA dimension measures feelings of competence and success in the workplace. Authorization for the questionnaire's reproduction and administration was granted by the publisher Mind Garden, that holds the instrument's copyrights. The punctuation for the analysis of the MBI-HSS was carried out through a 0 (never) to 6 (daily) scale. Scores for each dimension were calculated, and cut-off scores indicating abnormal results were obtained in accordance with Benevides-Pereira (20). As an indicative of burnout diagnosis, were followed the following criteria encompassing high EE (>26 points), high DE (>9 points), and low PA (<33 points) (21, 22).

### Procedures

To recruit the research's participants, doctors who had been working in patient assistance in the same institution for at least three uninterrupted months and who were not off work at the moment of the collection were considered eligible. The research instruments and informed consent forms were provided along with information on the research goals. Participants indicated the most convenient time and location for data collection, and psychology professionals were included in the stages of data collection and analysis. Data was obtained directly from the participants, who responded to the measures using pen and paper and were able to solve any questions with the data collection team at any time. All the stages of this research took place between January 2019 to March 2020.

The current research followed the Resolution n. 466/2012/CNS/MS/CONEP and it has been approved by the Research Ethics Committee of the Western Paraná State University, as demonstrated in Decision n. 3.200.235. Moreover, based on Cohen's *f* ^2^, a power of 98% and a 0.05 alpha was achieved.

### Data Analysis

Relative and absolute frequencies (categorical variables) were used to characterize the sample along with means and standard deviations (continuous variables). The normality of the data distribution and the homogeneity of variances were tested with the Kolmogorov-Smirnov and the Levene tests, respectively. The three domains of burnout were considered as dependent (i.e., outcome) variables for the analyses. Besides, a general score was created for the syndrome, using the following operation: emotional exhaustion + depersonalization—personal accomplishment (21, 22); this score was also used as an outcome variable. Comparisons between the independent variables general (composite) scores and the specific domains were carried out with Student's *t*-test for independent variables and univariate analysis of variance, besides the Mann-Whitney and Kruskal-Wallis *U*-tests. Exposure variables are included in Table 1. For the multivariate models, variables that presented *p* <0.20 in the unadjusted analysis were taken to simple linear regression models and consisted of our predictors, but only variables with *p* < 0.05 after the adjustment by independent variables continued in the final models. The rule of thumb for variable selection for regression analyses (i.e., *p* <0.20) was based on the principles of parsimony, exclusion of negligible/confounder effects, and the stopping rule (23). Variables from the multivariate procedures were labeled as risk factors when they were associated to negative outcomes (such as higher BS, higher EE, and higher DE, or with low PA). All analyses were carried out in SPSS 25.0.

**Table 1:** General characteristics of the sample.

	***n***	**%**
**Sex**		
Male	89	73.6
Female	32	26.4
**Age**		
25–39	57	50.4
40–69	56	49.6
**Civil state**		
Married	86	71.1
Singles/divorced/widower	35	23.1
**Children**		
Yes	77	63.6
No	44	36.4
**Year of graduation**		
1979–2004	57	48.3
2005–2018	61	51.7
**Number of workplaces**		
1–3	63	52.5
4 or more	57	47.5
**Weekly workload**		
40–60 h	60	49.6
70 h or more	61	50.4
**Type of work contract**		
Outsourced	92	77.3
Other	27	22.7
**Night shifts**		
Yes	98	81.0
No	23	19.0
**Patients seen per day**		
Up to 25	69	57.0
25 or more	52	43.0
**Teaching activity**		
Yes	36	29.8
No	85	70.2
**Monthly income**		
R$3.000–R$27.000	44	48.9
R$28.000 or more	46	51.1
**Physical exercise**		
Yes	82	68.3
No	38	31.7
**Time passed since the last vacations**		
<6 months	58	48.7
More than 6 months	61	51.3
**Satisfaction with the institution**		
Satisfied/very satisfied	72	60.5
Indifferent/not satisfied/very unsatisfied	47	39.5
**Diagnosis of psychological disorder**		
Yes	24	20.0
No	96	80.0
**Use of psychiatric medication**		
Yes	26	21.7
No	94	78.3
**Seeing psychologist/psychiatrist**		
Yes	30	25.0
No	90	75.0
**Clinical/chronic pathologies**		
Yes	43	36.4
No	75	63.6
**Contemplates changing careers**		
Yes	30	25.0
No	90	75.0
**Predominant area of work**		
ICU/Surgery	10	8.3
Emergency	25	20.8
Others	85	70.8
**Role in the institution**		
Routine doctor and clinician	42	35.0
On-call doctor	70	58.3
Administrative and head of clinical workers	8	6.7
**Medical specialty**		
Anaesthesiology	9	8.0
Cardiology	8	7.1
Pediatrics	8	7.0
Intensive medicine	7	6.2
Gynecology and obstetrics	19	16.8
Orthopedics and traumatology	13	11.5

*The data are expressed in absolute values and percentages*.

## Results

Table 1 presents the general characteristics of the sample. As to the profiles of the respondents, the majority was male (*n* = 89; 73.6%). Their ages ranged from 25 to 69 years and the mean age was 40.89 years (SD = 10.13). Most were Brazilian (99.2%), 71.1% were married, 23.1% were single, and 5.8% were divorced or widowers. The average number of kids they had was 1.16 (SD = 1.17), varying from 0 to 5 children. About half of them reported working more than 70 h a week and not having vacations in the last 6 months. The frequency with which they presented diagnoses of psychological disorders, use of medications, seeing a psychiatrist, and intention to change careers was between 20 and 25%.

Using criteria to detect alterations in the three dimensions of BS, we identified a general prevalence of 7.5% (95% CI: 3.8–13.8). Moreover, 33.9% (95% CI: 26.0–42.7) had high higher scores for EE, 31.4% (95% CI: 23.8–40.2) for high DE, and 20.8% (95% CI: 14.5–29.0) for low PA. The comparisons between the general scores and each dimension regarding the independent variables are presented in Table 2. The lowest EE values were observed among the oldest people (*p* = 0.02), with a workload of 40–60 h a week (*p* = 0.001), who do not do night shifts (*p* = 0.002), who do physical exercise (*p* = 0.016), took vacations <6 months ago (*p* = 0.001), feel satisfied with the institution they currently work in (*p* < 0.001), do not have a diagnosis of any psychological disorder (*p* = 0.01), did not consult a psychiatrist (*p* = 0.003), and did not consider changing careers (*p* < 0.001). Those who work longer shifts (*p* = 0.004), see more than 25 patients a day (*p* = 0.04), are dissatisfied with their current institutions (*p* < 0.001), and considered changing careers (*p* < 0.001) present larger DE values. Greater PA values were observed among participants with lighter workloads (*p* = 0.003), who were not outsourced workers (*p* = 0.012), do not do night shifts (*p* = 0.001), who do physical activities (*p* = 0.043), took vacations in the last 6 months (*p* = 0.004), are satisfied with the institution they work in (*p* < 0.001), and did not consider changing careers (*p* = 0.002). Finally, improved indicators of BS's general score (negative values) were observed for those with reduced workloads (*p* < 0.001), who do physical exercises (*p* = 0.015), took vacations in the last 6 months (*p* < 0.001), are satisfied with the institution they work in (*p* < 0.001), do not have a mental disorder diagnosis (*p* = 0.027), did not see psychiatrists (*p* = 0.007), and did not consider changing careers (*p* < 0.001).

**Table 2:** Scores of specific dimensions and general score of Burnout Syndrome in doctors.

	**EE**	**DE**	**PA**	**Total**
Total	21.4 ± 12.0	7.1 ± 6.1	38.1 ± 6.8	−9.4 ± 20.4
**Sex**				
Male	20.8 ± 11.6	6.9 ± 6.0	38.4 ± 6.9	−10.4 ± 19.7
Female	23.1 ± 13.0	7.7 ± 6.5	37.4 ± 6.6	−6.6 ± 22.2
*p*-value	0.326	0.578	0.404	0.391
**Age**				
25–39	23.8 ± 11.7	7.5 ± 5.9	37.5 ± 5.9	−6.2 ± 19.5
40–69	18.7 ± 12.1	6.4 ± 6.1	38.6 ± 7.5	−13.2 ± 21.0
*p*-value	0.020	0.356	0.166	0.071
**Civil state**				
Married	22.2 ± 11.6	7.3 ± 6.4	38.3 ± 6.9	−8.9 ± 20.8
Single/divorced/widower	19.5 ± 12.8	6.8 ± 5.2	37.6 ± 6.7	−10.8 ± 19.5
*p*-value	0.503	0.663	0.760	0.637
**Children**				
Yes	21.1 ± 12.1	6.7 ± 5.9	38.4 ± 7.4	−10.6 ± 20.9
No	21.9 ± 11.9	7.9 ± 6.4	37.6 ± 5.7	−7.3 ± 19.6
*p*-value	0.704	0.316	0.281	0.398
**Year of graduation**				
1979–2004	19.5 ± 11.9	7.1 ± 6.2	38.6 ± 7.8	−11.7 ± 20.9
2005–2018	23.4 ± 11.7	7.2 ± 6.1	37.8 ± 5.8	−7.1 ± 19.5
*p*-value	0.067	0.864	0.183	0.223
**Number of workplaces**				
1–3	20.6 ± 11.3	6.7 ± 5.5	38.8 ± 6.8	−11.3 ± 19.1
4 or more	22.3 ± 12.8	7.5 ± 6.7	37.5 ± 6.8	−7.7 ± 21.8
*p*-value	0.525	0.491	0.237	0.340
**Weekly workload**				
40–60 h	18.0 ± 12.3	5.6 ± 5.5	39.8 ± 6.7	−15.9 ± 20.4
70 h or more	24.7 ± 10.7	8.7 ± 6.3	36.5 ± 6.6	−3.1 ± 18.5
*p*-value	0.001	0.006	0.003	<0.001
**Type of work contract**				
Outsourced	21.9 ± 12.0	7.3 ± 6.0	37.5 ± 6.7	−8.0 ± 19.7
Others	19.2 ± 12.3	6.2 ± 6.5	40.9 ± 6.4	−15.5 ± 21.9
*p*-value	0.271	0.403	0.012	0.092
**Night shifts**				
Yes	22.9 ± 11.7	7.5 ± 6.0	37.2 ± 6.9	−7.4 ± 20.0
No	15.0 ± 11.4	6.4 ± 6.3	42.1 ± 4.8	−13.1 ± 20.9
*p*-value	0.002	0.312	0.001	0.143
**Patients seen per day**				
Up to 25	20.2 ± 11.8	6.2 ± 5.6	38.8 ± 6.0	−12.2 ± 18.2
25 or more	23.0 ± 12.1	8.5 ± 6.5	37.2 ± 7.6	−5.8 ± 18.2
*p*-value	0.203	0.039	0.389	0.088
**Teaching activity**				
Yes	20.0 ± 11.6	6.6 ± 6.1	39.4 ± 6.4	−12.2 ± 19.8
No	22.0 ± 12.2	7.4 ± 6.1	37.6 ± 7.0	−8.3 ± 20.7
*p*-value	0.411	0.551	0.176	0.340
**Monthly income**				
R$3.000–R$27.000	20.7 ± 12.9	6.2 ± 6.2	37.7 ± 6.8	−10.5 ± 21.1
R$28.000 or more	22.1 ± 11.8	8.3 ± 5.9	38.0 ± 6.7	−7.6 ± 19.3
*p*-value	0.523	0.108	0.941	0.509
**Physical exercise**				
Yes	19.6 ± 10.5	6.9 ± 5.9	39.0 ± 6.1	−12.2 ± 17.6
No	25.7 ± 13.6	7.7 ± 6.4	36.0 ± 7.7	−2.6 ± 23.7
*p*-value	0.016	0.527	0.043	0.015
**Time passed since the last vacations**				
<6 months	17.7 ± 9.3	6.2 ± 4.8	40.0 ± 6.2	−15.8 ± 15.7
More than 6 months	25.1 ± 13.0	8.0 ± 7.0	36.3 ± 7.0	−3.2 ± 21.9
*p*-value	0.001	0.096	0.004	<0.001
**Satisfaction with the institution**				
Satisfied/very satisfied	17.5 ± 10.9	5.1 ± 4.8	40.2 ± 6.2	−17.4 ± 17.1
Indifferent//not satisfied/very unsatisfied	27.7 ± 10.9	10.3 ± 6.5	34.6 ± 6.4	3.3 ± 18.2
*p*-value	<0.001	<0.001	<0.001	<0.001
**Diagnosis of psychological disorder**				
Yes	27.3 ± 13.2	8.6 ± 7.4	37.0 ± 7.1	−1.1 ± 21.7
No	20.0 ± 11.3	6.9 ± 5.7	38.4 ± 6.8	−11.4 ± 19.7
*p*-value	0.010	0.298	0.438	0.027
**Use of psychiatric medications**				
Yes	25.3 ± 13.1	8.2 ± 7.2	35.9 ± 7.5	−2.4 ± 21.8
No	20.3 ± 11.6	6.9 ± 5.7	38.8 ± 6.6	−11.3 ± 19.8
*p*-value	0.086	0.373	0.114	0.050
**Seeing a psychologist/psychiatrist**				
Yes	27.6 ± 13.7	8.8 ± 7.8	37.1 ± 6.7	−0.7 ± 23.3
No	19.3 ± 10.8	6.7 ± 5.3	38.5 ± 6.9	−12.3 ± 18.7
*p*-value	0.003	0.172	0.299	0.007
**Clinical/chronic pathologies**				
Yes	20.7 ± 11.5	6.7 ± 6.2	38.2 ± 7.6	−10.3 ± 19.4
No	21.9 ± 13.0	7.5 ± 6.1	38.0 ± 6.5	−8.5 ± 21.3
*p*-value	0.587	0.466	0.686	0.657
**Contemplates changing profession**				
Yes	32.1 ± 10.8	11.9 ± 7.6	35.0 ± 6.3	9.0 ± 17.9
No	17.8 ± 10.2	5.6 ± 4.6	39.2 ± 6.7	−15.5 ± 17.4
*p*-value	<0.001	<0.001	0.002	<0.001
**Predominant area of work**				
ICU/Surgery	24.5 ± 10.5	9.1 ± 6.2	37.1 ± 3.9	−0.8 ± 15.6
Emergency/Others	22.4 ± 12.9	9.0 ± 6.0	37.9 ± 6.9	−6.4 ± 19.5
*p*-value	0.503	0.073	0.594	0.231
**Role in the institution**				
Routine doctor and clinician	19.3 ± 12.0	6.3 ± 6.3	39.3 ± 6.7	−13.3 ± 21.4
On-call doctor	22.5 ± 12.5	7.6 ± 6.0	37.4 ± 6.7	−7.3 ± 20.0
Administrative and head of clinical workers	22.5 ± 6.9	6.9 ± 5.7	39.4 ± 7.8	−10.0 ± 19.1
*p*-value	0.285	0.530	0.285	0.329

*DE, Despesonalization; EE, Emotional exhaustion; PA, Personal Accomplishment; BS, Burnout Syndrome. The data are expressed as means and standard deviations. P-values were obtained with t-tests for independent samples and univariate ANOVA (total score) tests, along with U-tests by Mann-Whitney and Kruskal-Wallis*.

The independent variables with *p*-values below 0.20 were taken to the linear regression models (Table 3) for examining the associated risk and protective factors. After constructing the final models, higher explanation coefficients (*R*^2^) were detected for EE and for BS's general score. Three variables independently explained DE and PA, while four other variables composed the BS general score model, and five variables composed the model for EE. The main associated factors were satisfaction with the institution (found in all models) and intent to change careers (in the models for EE, DE, and PA). Consulting psychiatrists and practicing physical activities were factors retained in the models for EE and BS. Sociodemographic and work-related variables present solely in one of the models were: working night shifts (PA), length of the last vacations (PA), number of patients a day (DE), and age (EE).

**Table 3:** Factors associated with BS in doctors in doctors in Paraná, Brazil.

	**β (95% CI)**	***P***	**β_ADJ_ (95% CI)**	***p***
**Emotional exhaustion**			***R*** **^2^ = 0.45**	
Age	−5.13 (−9.56; −0.70)	0.024	−4.12 (−7.59; −0.66)	0.020
Year of graduation	3.89 (−0.43; 8.20)	0.077	—	—
Workload	6.66 (2.49; 10.82)	0.002	—	—
Night shifts	−7.89 (−13.23; −2.55)	0.004	—	—
Physical exercise	6.10 (1.60; 10.61)	0.008	4.58 (0.89–8.28)	0.015
Time passed since the last vacations	7.39 (3.29; 11.50)	0.001	—	—
Satisfaction with the institution	10.18 (6.14; 14.22)	<0.001	7.67 (4.07; 11.27)	<0.001
Diagnosis of psychological disorder	−7.42 (−12.71; −2.12)	0.006	—	—
Use of psychiatric medications	−4.99 (−10.22; 0.24)	0.061	—	—
Seeing a psychologist/ psychiatrist	−8.22 (−13.05; −3.40)	0.001	−4.68 (−8.74; −0.62)	0.024
Contemplates changing careers	−14.31 (−18.64; −9.99)	<0.001	−11.55 (−15.71; −7.38)	<0.001
**Depersonalization**			***R*** **^2^ = 0.32**
Workload	3.03 (0.91; 5.17)	0.006	—	—
Patients seen per day	2.30 (0.12; 4.49)	0.039	2.61 (0.75; 4.45)	0.007
Monthly income	2.08 (−0.46; 4.62)	0.108	—	—
Time passed since last vacations	1.84 (−0.35; 4.03)	0.098		
Satisfaction with the institution	5.20 (3.14; 7.27)	<0.001	3.68 (1.73; 5.64)	<0.001
Contemplates changing careers	−6.21 (−8.50; −3.93)	<0.001	−5.25 (−7.45; −3.05)	<0.001
Predominant area of work	−1.86 (-3.56; −0.15)	0.034	—	—
**Personal accomplishment**			***R*** **^2^ = 0.22**	
Age	1.09 (−1.45; 3.63)	0.396	—	—
Year of graduation	−0.82 (−3.31; 1.67)	0.516	—	—
Workload	−3.27 (−5.68; −0.87)	0.008	—	—
Type of work contract	3.41 (0.53; 6.28)	0.021	—	—
Night shifts	4.97 (1.95; 7.98)	0.001	3.69 (0.86–6.53)	0.011
Teaching activities	−1.77 (−4.48; 0.935)	0.197	—	—
Doing physical exercise	−2.95 (−5.55; −0.35)	0.027	—	—
Time passed since last vacations	−3.62 (−6.02; −1.22)	0.003	−2.42 (−4.67 to 1.64)	0.003
Satisfaction with the institution	−5.58 (−7.92; −3.26)	<0.001	−4.58 (−6.90 to 2.26)	<0.001
Use of psychiatric medications	2.87 (−0.11; 5.85)	0.059	—	—
Contemplates changing careers	4.18 (1.41; 6.95)	0.003	—	—
**Burnout syndrome**			***R*** **^2^ = 0.46**	
Age	−6.97 (−14.56; −0.62)	0.071	—	—
Workload	12.71 (5.68; 19.75)	<0.001	—	—
Type of work contract	−7.53 (−16.30; 1.24)	0.092	—	—
Night shifts	−5.68 (−13.29; 1.94)	0.143	—	—
Patients seen per day	6.40 (−0.98; 13.78)	0.088	—	—
Doing physical activities	9.62 (1.93; 17.31)	0.015	7.39 (1.49; 13.28)	0.014
Time passed since the last vacations	12.60 (5.60; 19.59)	0.001	—	—
Satisfaction with the institution	20.77 (14.23; 27.32)	<0.001	16.16 (10.29; 22.04)	<0.001
Diagnosis of psychological disorder	−10.31 (-19.41; −1.20)	0.027	—	—
Use of psychiatric medications	−8.87 (−17.75; 0.02)	0.050	—	—
Seeing a psychologist/psychiatrist	−11.57 (−19.90; −3.24)	0.007	−8.99 (−15.42; −2.55)	0.007
Contemplates changing careers	−24.54 (−31.87; −17.21)	<0.001	−17.15 (-23.84; −10.45)	<0.001

*R^2^, coefficient of determination. The unadjusted models are composed by the variables that presented p <0.20 in bivariate analysis. Only the coefficient of variables that obtained p <0.05 are presented in the adjusted analysis. Data are presented in unadjusted and adjusted coefficients, and with 95% confidence intervals (linear regression)*.

## Discussion

The current investigation builds upon past research and provides an updated understanding of the BS in the studied sample while enriching the literature with recent data on factors underpinning MDs experiences with the burnout syndrome. The relatively low prevalence of BS (7.5%) in the present study might be partially explained by cultural differences and by the the adoption of the Maslach criteria, considered a rigorous set of rules that contemplate the relation of the syndrome's three dimensions (9). In general, the MBI-HSS results in 2.6–11.8% prevalence ratings among healthcare professionals in different specialties and regions of the world (24). In Brazil, the study of (25) found a 5.1% prevalence of the syndrome among doctors in the city of Recife (11); reported a 10.4% prevalence among anesthesiologists. Magalhães and Glina (26) found a 11% prevalence of BS in doctors in the state of São Paulo, while Barros et al. (27) reported a 42.6% prevalence among intensive care doctors in the state of Sergipe. Moreover, research adopting other criteria that does not contemplate the multidimensionality of BS might indicate a prevalence of the syndrome among doctors of up to 80.5% (24).

Our findings related to the proportion of participants who had high scores in EE (33.9%) and DE (31.4%) show larger numbers than the data presented by Maticorena-Quevedo et al. (28) for Peruvian doctors, as well as the one obtained by Magalhães et al. (11) for anesthesiologists in Brazil's Federal District. The results of Barros et al. (27) for intensive care doctors in the state of Sergipe showed larger EE (66.4%) and DE (54.9%), and the same applies for the data presented by Lima et al. (25), which result from a research in the city of Recife (EE = 61.0%; DE = 36.7%). These findings are worrisome, as BS develops with time, making the ailment invisible for a while (25). The main risk factors associated with EE were related to the excessive workload and with the desire to change careers, corroborating the study's hypothesis and also in line with previous findings (11, 13). One central aspect of BS is elevated EE. That occurs due to the workers' draining of emotional resources (4), which interferes with their sense of achievement and cause them to question their careers. There is a consensus as to the negative consequences of EE to the doctor, patient, and health system in general (14).

Advanced age might be considered a protective factor for EE based on to the understanding that, over time, doctors might manage both stressful situations and emotional reactions in a more effective way, probably scaffolded by their work experience (29). In this sense, they are different from younger doctors, who are not as prepared to deal with emotional conflicts and have less experience controlling their feelings, thus facing difficulties in dealing with pressure situations (15, 30). In the bibliographic review, no consensus on the role of advanced age in BS and its dimensions was found. In some works, age operates as an unleashing factor for BS (7), though other studies have not identified any associations (9, 31). In our investigation, age was a significant protective factor for EE, thus representing that older individuals had lower odds of presenting with elevated EE scores.

Another hypothesis of the study referred to the support provided by mental health professionals. Indeed, consulting with psychologists and psychiatrists is apparently a moderating factor between work demands and BS, and doctors who sought these professionals presented lower levels of EE. The relation between psychosocial support and easier adaptation to the work dynamics and personal relations supports is an explanation for that. Social support through psychotherapy is important for the well-being, physical and mental health of the worker, contributing positively to the reduction of BS (16). Individual interventions produce short-term benefits (6 months), while organizational ones have longer-term (>1 year) advantages in reducing BS (14).

Statistical associations between high DE and seeing more than 25 patients a day, dissatisfaction with the institution, and considering changing careers might reflect feelings of disappointment, frustration, as well as of not having the required energy to dispose for the work. Hence, the professional seeks to distance him or herself from the receivers of the service, actively ignoring the qualities that make them unique and involving. Dissatisfaction with the institution is related to the style of the supervision doctors are subjected to, the physical work environment, the hierarchies in place, benefits, and hospital policies established for the clients (and not workers), thus engendering negative tensions between those who execute the work and those who receive it (15).

Detachment is a defensive immediate reaction following exhaustion (11, 13). It demonstrates that the work demands a significant effort from the professional. Doctors' relations in the hospital environment involve people—many of them, in critical situations. It is expected that these professional comfort patients and deal with the frustrations related to the limitations of medicine toward disease (9, 25). These elements have been associated to EE and DE (15).

We identified high levels of PA (79.8%), although in a lesser proportion than reported by Lima et al. (25) (81%), and also larger than the one found by Maticorena-Quevedo et al. (28) (18.1%), Barros et al. (27) (67.2%), and Magalhães et al. (11) (47.7%). In this sense, a recently published systematic review evaluated 182 studies involving 109.628 doctors and pointed to a global prevalence of BS between 0 and 87.1% (24). Studies suggest differences in the level of PA between the sexes; that has not been identified in the present research. We observed that the professionals who do not do night shifts, who took vacations in the last 6 months, and who are satisfied with the institutions they work in presented higher PA scores. That is possibly related to stress relief, changing environments, and the enlarged possibility of leisure and family times. Grover et al. (14) highlight that longer vacations reduce the syndrome's incidence; similarly, the work overload related to night shifts increases the chances of the syndrome (12). High PA indicates that the worker is happy with his or her own work and performance (15), which probably mitigates the consequences of high levels of EE and DE, reducing the chances of the syndrome (25). In this study, factors like physical exercise, satisfaction with the institution, seeing a psychologist or psychiatrist, and cogitation of changing professions were kept in the multivariate model for BS. Notwithstanding studies with healthcare professionals' pointing to the positive effects of physical exercise for reducing burnout (32, 33), a recent meta-analysis has not established a generalized supposition that exercise is a successful tool to relieve BS symptoms (34). These results must be treated with caution, as the meta-analysis included only four studies, with a total sample of 248 participants.

Other studies concluded that regular, intense physical exercise promotes positive changes relevant for the prevention of burnout (35, 36), that factor deserving improved attention from researchers (i.e., randomized clinical trials or studies with robust methodological designs) (34). The effects of physical activity might be associated with psychological alterations promoted by the changes in environment, behavior distraction, reducing anxiety, strengthening friendship bounds, and physiological alterations that reduce people's sensitivity to fatigue and promote general improvements in global physical health (34, 35).

Data related to satisfaction with the institution corroborate previous statements in the literature, according to which the work environment (occupational stress and work conditions) is one of the factors influencing workers' health (15, 37, 38). Dissatisfaction with the institution is an important factor for EE, involving not only the physical environment, but also the hierarchical relations in place, opportunities of growth, and perceptions of the work itself (13, 39, 40). Strategies to promote changes in the work environment might include the institutional valorization and support of the doctor, adequate career management, incentives for permanent education, innovation, stimulus for autonomy, strengthening personal relations among colleagues, superiors, and clients, among other things (41). Other studies also found associations between burnout and contemplating giving up the profession, as we have in the present investigation (42, 43). Resistance among doctors to seek professional help is remarkable, as they understand that psychological support would weaken their professional role and place them in a vulnerability situation, and do not concede that such a support might cushion the negative effects of burnout (14).

The study also had some limitations to be taken into account in the analysis of its conclusions. First, we highlight its cross-sectional design, which hinders causal conclusions. Sample size was also quite modest in comparison to most epidemiological investigations on the matter, which could have implications in terms of generalization of the study's findings. Importantly, even though efforts to deal with bias were carried out (i.e., inclusion of psychology professionals to assist in data collection and specification of MD who had been working in the same institution uninterruptedly for at least 3 months), one should also consider the impact of healthy workers in the results—a peculiar issue in cross-sectional epidemiological studies that frequently exclude sick individuals (44). Many times, as the professionals are no longer able to continue working, they begin taking leaves for health treatments.

Additionally, some sort of bias related to depersonalization is also plausible, as it is difficult for workers to admit practicing emotional detachment and impersonal treatment toward patients (45). Findings related to age must be seen with caution, as there might exist the bias of survival—that is, that those who felt fatigued left the job early on, leaving behind survivors who, consequently, will display lower levels of the syndrome (29). Moreover, results obtained in this study cannot be generalized since Francisco Beltrão has a demographic profile that closely resembles the Southern Brazilian states, including a higher density of MD per 1,000 inhabitants; consequently, inferences to Northern regions of the country are not possible since cultural differences could drive significant differences in individual's self-reporting of their psychological symptoms (46). These sources of bias and limitations must be considered in the interpretation of the results.

In conclusion, factors more clearly associated with BS and its dimensions have been related to the satisfaction with the organization, the intention of changing careers, as well as to age and how the professionals face stress (i.e., seeing mental health specialists or physical exercise). As discussed, the risk and protective factors identified have some overlap with previous research, including the overall prevalence of the BS, which indicates to a possible tentative of generalization to the broader population of MD from similar contexts (14, 25, 36, 39, 42). Additionally, the study presents data that might be useful in planning intervention mechanisms to improve management and decision-making within policies for workers. We emphasize the need for investments in policies for human resources in hospitals, especially in actions seeking to reduce pressures generating work stress, which improve the organizational processes and make a more adequate support to work and the medical worker.

## Data Availability Statement

The raw data supporting the conclusions of this article will be made available by the authors, without undue reservation.

## Ethics Statement

The studies involving human participants were reviewed and approved by Western Paraná State University Research Ethics Committee. The patients/participants provided their written informed consent to participate in this study.

## Author Contributions

NB, LF, and JA contributed to conception and design of the study. NB, LF, and GW acquired and organized the data. NB, JA, LF, and AC performed the analysis and interpretation of data. NB, JA, and GW wrote the first draft of the manuscript. AC, JA, and FF wrote sections of the manuscript. LF, FF, GW, JA, and AC contributed to the manuscript final version. All authors contributed to the article and approved the submitted version.

## Conflict of Interest

The authors declare that the research was conducted in the absence of any commercial or financial relationships that could be construed as a potential conflict of interest.

## Publisher's Note

All claims expressed in this article are solely those of the authors and do not necessarily represent those of their affiliated organizations, or those of the publisher, the editors and the reviewers. Any product that may be evaluated in this article, or claim that may be made by its manufacturer, is not guaranteed or endorsed by the publisher.

## References

[1] Campos JF David HSL. Avaliação do contexto de trabalho em terapia intensiva sob o olhar da psicodinâmica do trabalho. Rev Escola Enfermagem USP. (2011) 45:363–8. 10.1590/S0080-62342011000200009 21655785

[2] Campos ICM Angélico AP Oliviera MS Oliveira DCR. Fatores sociodemográficos e ocupacionais associados à síndrome de burnout em profissionais de enfermagem. Psicol Reflexão Crí*tica*. (2015) 28:764–71. 10.1590/1678-7153.201528414

[3] Amariz AA Paula AC Rosário BC Gitirana B Rosado GT Ribeiro F. Prevalência da síndrome de burnout em médicos e médicos residentes em montes claros-MG no ano de 2014. Rev Unimontes Cientí*fica*. (2016) 18:62–75.

[4] Maslach C Jackson SE. The measurement of experienced burnout. J Occup Behav. (1981) 2:99–113. 10.1002/job.403002020525855820

[5] Morelli SGSM Sapede M Silva ATC. Burnout em médicos da Atenção Primária: uma revisão sistemática. Rev Brasil Med Famí*l Comun*. (2015) 10:1–9. 10.5712/rbmfc10(34)958

[6] Barbosa G Andrade E Carneiro M Gouveia V Barbosa EA Santos RJE. A Saúde dos Médicos no Brasil. Brasília: Conselho Federal de Medicina (2007).

[7] Lima CR Sepúlveda JL Lopes PH Fajardo HD Sousa MM Júnior MC. Prevalência da síndrome de Burnout em médicos militares de um hospital público no Rio de Janeiro. Rev Brasil Med Trabalho. (2018) 16:287–96. 10.5327/Z1679443520180297 32270089 PMC7104827

[8] Teixeira F Silva M Medeiros G. Síndrome de Burnout: a interface entre o trabalho na área de educação e na enfermagem. Rev Enferm Ref. (2010) 3:101–19. 10.12707/RII0931

[9] Moreira HA Souza KN Yamaguchi MU. Síndrome de burnout em médicos: uma revisão sistemática. Rev Brasil Saúde Ocup. (2018) 43:1–11. 10.1590/2317-6369000013316

[10] Jarruche LT Mucci S. Síndrome de burnout em profissionais da saúde: Revisão integrativa. Rev Bio. (2021) 29:162–73. 10.1590/1983-8042202129145628977238

[11] Magalhães E Oliveira ÁC Govêia CS Ladeira LC Queiroz DM Vieira CV. Prevalência de síndrome de burnout entre os anestesiologistas do distrito federal. Rev Brasil Anestesiol. (2015) 65:104–10. 10.1016/j.bjan.2013.07.016 25740276

[12] Barros DD Tironi MO Nascimento Sobrinho CL Neves FS Bitencourt AG. Médicos plantonistas de unidade de terapia intensiva: Perfil sócio-demográfico, condições de trabalho e fatores associados à síndrome de burnout. Rev Brasil Terapia Int. (2008) 20:235–40. 10.1590/S0103-507X2008000300005 25307090

[13] Sá AMS Martins-Silva PO Funchal B. O impacto da satisfação no trabalho em profissionais de enfermagem. Psicol Soc. (2014) 26:664–74. 10.1590/S0102-7182201400030001528591294

[14] Grover S Adarsh H Naskar C Varadharajan N. Physician burnout: a review. J Mental Health Human Behav. (2018) 23:78–85. 10.4103/jmhhb.jmhhb_47_19

[15] Rosa C Carlotto MS. Síndrome de burnout e satisfação no trabalho em profissionais de uma instituição hospitalar. Rev SBPH. (2005) 8:1–15.

[16] Andrade T Hoch REE Vieira KM Rodrigues CMC. Síndrome de burnout e suporte social no trabalho: a percepção dos profissionais de enfermagem de hospitais públicos e privados. Organ Soc. (2012) 19:231–51. 10.1590/S1984-92302012000200004

[17] Campos ICM. Fatores Sociodemográficos e Ocupacionais Associados à Síndrome de Burnout em Profissionais de Enfermagem. Brasil: Dissertação de mestrado, Universidade Federal de São João del-Rei, São João del-Rei (2013).

[18] Lautert L. O desgaste profissional do enfermeiro. Espanha: Tese, Universidade Pontifica de Salamanca (1995).

[19] Rodriguez LCA Estigarribia G Guerrero C. Prevalencia de síndrome de burnout em médicos del hospital regional de coronel oviedo. Rev Cient Facult Cien Med. (2018) 1:44–53.

[20] Benevides-Pereira AMT. Burnout: Quando o Trabalho a Saúde Ameaça o Bem-Estar do Trabalhador. 1ª ed. São Paulo: Casa do Psicólogo (2002).

[21] Ramirez AJ Graham J Richards MA Cull A Gregory WM Leaning MS. Burnout and psychiatric disorder among cancer clinicians. Brit J Cancer. (1995) 71:1263–9. 10.1038/bjc.1995.244 7540037 PMC2033827

[22] da Silva DK Pacheco MD Marques HS Branco RC da Silva MA Nascimento MD. Burnout no trabalho de médicos pediatras. Rev Brasil Med Trabalho. (2017) 15:2–11. 10.5327/Z1679443520177032

[23] Chowdhury MZI Turin TC. Variable selection strategies and its importance in clinical prediction modelling. Family Med Commun Health. (2020) 8:e000262. 10.1136/fmch-2019-000262 32148735 PMC7032893

[24] Rotenstein LS Torre M Ramos MA Rosales RC Guille C Sem S. Prevalence of burnout among physicians. JAMA. (2018) 320:1131–50. 10.1001/jama.2018.12777 30326495 PMC6233645

[25] Lima RAS Souza AI Galindo RH Feliciano KVO. Vulnerabilidade ao burnout entre médicos de hospital público do recife. Ciência Saúde Colet. (2013) 18:1051–8. 10.1590/S1413-81232013000400018 23670382

[26] Magalhães RAC Glina DMR. Prevalência de burnout em médicos de um Hospital Público de São Paulo. Rev Saúde Ética Justiça. (2006) 11:29–35. 10.11606/issn.2317-2770.v11i1-2p29-35

[27] Barros MM Almeida SP Barreto AL Faro SR Araújo MRM Faro A. Síndrome de burnout em médicos intensivistas: estudo em UTIs de sergipe. Temas Psicol. (2016) 24:377–89. 10.9788/TP2016.1-26

[28] Maticorena-Quevedo JM Beas R Anduaga-Beramendi A Mayta-Tristán P. Prevalência del síndrome de burnout em médicos y enfermeras del perú, ensusalud 2014. Rev Peruana Med Exp Salud Publica. (2016) 33:241–7. 10.17843/rpmesp.2016.332.2170 27656922

[29] Maslach C Schaufeli WB Leiter MP. Job burnout. Ann Rev Psychol. (2001) 52:397–422. 10.1146/annurev.psych.52.1.397 11148311

[30] Benevides-Pereira AMT. Burnout: Quando o Trabalho Ameaça o Bem-Estar do Trabalhador 4th ed. 1 reimpressão. São Paulo: Casa do Psicólogo (2014).

[31] Aguilera EC García JE. Prevalencia del síndrome de agotamiento professional (Burnout) em médicos familiares mexicanos: análisis de factores de riesgo. Rev Colomb Psiquiatria. (2010) 39:67–84. 10.1016/S0034-7450(14)60237-7

[32] Bretland R Thorsteinsson E. Reducing workplace burnout: the relative benefits of cardiovascular and resistance exercise. PeerJ. (2015) 3:891. 10.7717/peerj.891 25870778 PMC4393815

[33] Mealer M Conrad D Evans J Jooste K Solyntjes J Rothbaum B. Feasibility and acceptability of a resilience training program for intensive care unit nurses. Am J Crit Care. (2014) 23:97–105. 10.4037/ajcc2014747 25362680

[34] Ochentel O Humphrey C Pfeifer K. Efficacy of exercise therapy in persons with Burnout. A systematic review and meta-analysis. J Sports Sci Med. (2018) 17:475–84. 30116121 PMC6090391

[35] Gerber M Lindwall M Lindegård A Börjesson M Jonsdottir IH. Cardiorespiratory fitness protects against stress-related symptoms of burnout and depression. Patient Educ Counsel. (2013) 93:146–52. 10.1016/j.pec.2013.03.021 23623176

[36] Naczenski LM Vries JD Hooff MLM Kompier MAJ. Systematic review of the association between physical activity and burnout. J Occup Health. (2017) 59:477–94. 10.1539/joh.17-0050-RA 28993574 PMC5721270

[37] Silva MA Argimon II Wendt GW. Insegurança no trabalho e sua relação com a saúde psicológica do trabalhador. Rev Soc Psicol Rio Grande Sul. (2012) 1:40–7.

[38] Silva MA Wendt GW Argimon II. As condições do trabalho e do relacionamento do casal são protetivas à saúde geral? Um estudo sobre o modelo de ajustamento diádico no Brasil. Barbarói. (2012) 37:30–45.

[39] Pêgo FPL Pêgo DR. Síndrome de burnout. Rev Brasil Med Trabalho. (2016) 14:171–6. 10.5327/Z1679-443520162215

[40] Wallace JE Lemaire JB Ghali WA. Physician wellness: a missing quality indicator. Lancet. (2009) 374:1714–21. 10.1016/S0140-6736(09)61424-0 19914516

[41] Oliveira AM Silva MT Galvão TF Lopes LC. The relationship between job satisfaction, burnout syndrome and depressive symptoms: an analysis of professionals in a teaching hospital in Brazil. Medicine. (2018) 97:e13364. 10.1097/MD.0000000000013364 30544404 PMC6310545

[42] Mata C Machado S Moutinho A Alexandra D. Prevalência de síndrome de burnout nos profissionais dos cuidados de saúde primários. Rev Portug Med Geral Familiar. (2016) 32:179–86.

[43] Suñer-Soler R Grau-Martín A Flichtentrei D Prats M Braga F Font-Mayolas S. The consequences of burnout syndrome among healthcare professionals in Spain and spanish speaking latin American countries. Burn Res. (2014) 1:82–9. 10.1016/j.burn.2014.07.004

[44] Michael MJ. Standardized mortality ratios and the “healthy worker effect”: scratching beneath the surface. J Occup Med. (1976) 18:165–8. 10.1097/00043764-197603000-00009 1255276

[45] Dias S Queirós C Carlotto MS. Síndrome de Burnout e fatores associados em profissionais da área da saúde: um estudo comparativo entre Brasil e Portugal. Aletheia. (2010) 43:4–21.

[46] Scheffer M. Demografia Médica no Brasil 2018. São Paulo: Departamento de Medicina Preventiva da Faculdade de Medicina da USP, Conselho Regional de Medicina do Estado de São Paulo e Conselho Federal de Medicina (2018).

